# Exogenous Ketone Supplements Reduce Anxiety-Related Behavior in Sprague-Dawley and Wistar Albino Glaxo/Rijswijk Rats

**DOI:** 10.3389/fnmol.2016.00137

**Published:** 2016-12-06

**Authors:** Csilla Ari, Zsolt Kovács, Gabor Juhasz, Cem Murdun, Craig R. Goldhagen, Andrew P. Koutnik, Angela M. Poff, Shannon L. Kesl, Dominic P. D’Agostino

**Affiliations:** ^1^Department of Molecular Pharmacology and Physiology, Hyperbaric Biomedical Research Laboratory, Morsani College of Medicine, University of South FloridaTampa, FL, USA; ^2^Department of Zoology, University of West HungarySzombathely, Hungary; ^3^Proteomics Laboratory, Eotvos Lorand UniversityBudapest, Hungary

**Keywords:** anxiety, exogenous ketone supplements, ketones, elevated plus maze, animal models

## Abstract

Nutritional ketosis has been proven effective for seizure disorders and other neurological disorders. The focus of this study was to determine the effects of ketone supplementation on anxiety-related behavior in Sprague-Dawley (SPD) and Wistar Albino Glaxo/Rijswijk (WAG/Rij) rats. We tested exogenous ketone supplements added to food and fed chronically for 83 days in SPD rats and administered sub-chronically for 7 days in both rat models by daily intragastric gavage bolus followed by assessment of anxiety measures on elevated plus maze (EPM). The groups included standard diet (SD) or SD + ketone supplementation. Low-dose ketone ester (LKE; 1,3-butanediol-acetoacetate diester, ~10 g/kg/day, LKE), high dose ketone ester (HKE; ~25 g/kg/day, HKE), beta-hydroxybutyrate-mineral salt (βHB-S; ~25 g/kg/day, KS) and βHB-S + medium chain triglyceride (MCT; ~25 g/kg/day, KSMCT) were used as ketone supplementation for chronic administration. To extend our results, exogenous ketone supplements were also tested sub-chronically on SPD rats (KE, KS and KSMCT; 5 g/kg/day) and on WAG/Rij rats (KE, KS and KSMCT; 2.5 g/kg/day). At the end of treatments behavioral data collection was conducted manually by a blinded observer and with a video-tracking system, after which blood βHB and glucose levels were measured. Ketone supplementation reduced anxiety on EPM as measured by less entries to closed arms (sub-chronic KE and KS: SPD rats and KSMCT: WAG/Rij rats), more time spent in open arms (sub-chronic KE: SPD and KSMCT: WAG/Rij rats; chronic KSMCT: SPD rats), more distance traveled in open arms (chronic KS and KSMCT: SPD rats) and by delayed latency to entrance to closed arms (chronic KSMCT: SPD rats), when compared to control. Our data indicates that chronic and sub-chronic ketone supplementation not only elevated blood βHB levels in both animal models, but reduced anxiety-related behavior. We conclude that ketone supplementation may represent a promising anxiolytic strategy through a novel means of inducing nutritional ketosis.

## Introduction

Anxiety disorders, such as generalized anxiety disorder, phobia and panic disorder, are the most prevalent type of mental disorders (Li, 2012). Anxiety can be associated with psychiatric morbidity, disability, increased healthcare burden and mortality in the general population (Teri et al., 1999). These symptoms can cause significant distress interfering with a person’s quality of life, while they commonly occur along with other mental or physical illnesses, which may mask anxiety symptoms or aggravate them. Some symptoms, like fear and worry, occur in all anxiety disorders including generalized anxiety disorders, panic disorder and social anxiety disorder (Stahl, 2003; Mula, 2013). Our knowledge relating to exact cause and pathomechanism(s) of anxiety disorders is far from complete; however, it is known that the amygdala is determinant in the experience of fear and anxiety by mediating the autonomic and endocrine responses through the output to the hypothalamus, and avoidance behavior through the output to the periaqueductal gray matter (Stahl, 2003; Engin and Treit, 2008; Li, 2012; Mula, 2013). Previous studies also show that serotonergic, glutamatergic as well as GABAergic system have a role in the regulation of anxiety (Nagy et al., 1979; Kakui et al., 2009; Li, 2012; Dias et al., 2013). Anxiety and depression are common problems affecting people with epilepsy and Alzheimer’s disease (AD), and can exacerbate symptoms of Glucose transporter type-1 deficiency syndrome (GLUT1 DS). Comorbidity between anxiety, depression and AD has been recognized (Teri et al., 1999; Hamid et al., 2011; Mula, 2013), and anxiety plays a key role in suicidality among patients with depression (Placidi et al., 2000). Interestingly, the same brain regions involved in a significant proportion of patients with focal epilepsy, such as the amygdala and the hippocampus, also play a key role in the neurobiology of anxiety (Li, 2012; Dias et al., 2013).

Anecdotal reports suggest that nutritional ketosis can promote a reduction in anxiety, although there is currently no convincing evidence to indicate that elevated ketone levels would reduce anxiety in humans (Ehrenreich, 2006). The elevation of ketones such as beta-hydroxybutyrate (βHB) and acetoacetate (AcAc) associated with nutritional ketosis causes a fundamental shift in metabolic physiology and brain neuropharmacology that is associated with preservation of brain homeostasis (Bough and Rho, 2007; Yudkoff et al., 2007; D’Agostino et al., 2013). Ketosis can be achieved by prolonged fasting (Owen et al., 1967) or by strict adherence to a ketogenic diet (KD), which is a metabolic-based therapy, a high-fat (70%–85% kcal) and low carbohydrate (3%–5%) diet (Kwiterovich et al., 2003; De Giorgis and Veggiotti, 2013). Maintaining long-term ketosis has proven to be beneficial in epileptic patients by decreasing the frequency and severity of seizures (Kossoff et al., 2011). Nutritional ketosis has also confirmed beneficial effects in animal models and human patients with AD, GLUT1 DS and cancer (Poff et al., 2013, 2014; Veggiotti and De Giorgis, 2014; Newport et al., 2015). GLUT1 DS results from impaired glucose transport into the brain (Klepper and Voit, 2002), however, ketones use another transporter to enter the central nervous system (CNS) providing an alternative source of fuel. Therefore, nutritional ketosis is used as a treatment option in GLUT1 patients (De Giorgis and Veggiotti, 2013) effectively correcting the impaired brain energy metabolism, reducing the frequency of the seizures (Leen et al., 2010) and improving behavior in autism spectrum disorder (Herbert and Buckley, 2013). Despite the proven and emerging therapeutic applications of the KD, many patients experience difficulties with compliance or experience a loss of effectiveness over time; therefore, new therapeutic strategies are needed.

The Wistar Albino Glaxo/Rijswijk (WAG/Rij) rat strain was originally developed as an animal model of human absence epilepsy, as the animals show spontaneous spike-wave discharges in the EEG (Coenen and Van Luijtelaar, 2003). Nevertheless, WAG/Rij rats are often used for investigation of different CNS diseases, such as anxiety, similar to Sprague-Dawley (SPD) rats by means of elevated plus maze (EPM; Sarkisova et al., 2003; Kovács et al., 2006, 2012, 2015; Sarkisova and Kulikov, 2006; Sarkisova and van Luijtelaar, 2011; Rebuli et al., 2015). EPM is a widely used behavioral assay for rodents, and it has been validated to assess the anxiety responses of rodents (Pellow et al., 1985; Walf and Frye, 2007). This test relies upon rodents’ proclivity toward dark enclosed spaces (approach) and an unconditioned fear of heights/open spaces (avoidance; Barnett, 1975; Walf and Frye, 2007). It is a widely-used animal model and investigation method of anxiety that is primarily sensitive to benzodiazepine-type anxiolytics (e.g., diazepam; Paslawski et al., 1996). Anti-anxiety behavior (increased open arm time and/or open arm entries) can be determined, which reflects the rodent’s preference for protected areas (e.g., closed arms) and their innate motivation to explore novel environments (Walf and Frye, 2007). Consequently, EPM assay on SPD and WAG/Rij rats is a suitable method for investigate the effect of ketone supplementation-evoked changes on anxiety level.

We hypothesized that ketone supplementation would decrease measures of anxiety-related behavior assessed with EPM behavioral assay in two rat strains. We have previously characterized the effects of ketone supplementation on blood ketone levels given *via* intragastric gavage (D’Agostino et al., 2013; Kesl et al., 2015) in rats and *via* chronic feeding mice (Poff et al., 2014). Ketone supplementation causes a rapid and sustained increase in blood ketone level, which may evoke anxiolytic effect by the increase of the GABAergic effects (Yudkoff et al., 2007; Li, 2012) or through numerous neuropharmacological pathways (Rho, 2015). To ensure that our results were not strain dependent, we assessed the effects of ketone supplementation on anxiety in SPD rats as well as in WAG/Rij rats, which have reduced activity of GABAergic system (Luhmann et al., 1995). Thus, the focus of this study was to test and determine the effects of several forms of ketone supplementation on anxiety-related behavior by using EPM behavioral assay in two rat strains. Exogenous ketone supplements were fed chronically to SPD rats and administered sub-chronically (gavage bolus) to SPD rats and WAG/Rij rats prior to assessment of anxiety measures.

## Materials and Methods

### Animals

Two months old male SPD (*n* = 87) and 8 months old male WAG/Rij (*n* = 32) rats were used in the experiments. The animals were housed at Department of Molecular Pharmacology and Physiology (Hyperbaric Biomedical Research Laboratory, Morsani College of Medicine, University of South Florida, Tampa, FL, USA) and the Department of Zoology (University of West Hungary, Savaria Campus, Szombathely, Hungary). Animals were kept in groups of 2–4 under standard laboratory conditions (12:12 h light-dark cycle, light was on from 08:00 AM to 08:00 PM) in air-conditioned rooms at 22 ± 2°C.

Animal treatment and measuring procedures were performed in accordance with the University of South Florida Institutional Animal Care and Use Committee (IACUC) guidelines (Protocol #0006R) and with the local ethical rules in accordance with the Hungarian Act of Animal Care and Experimentation (1998. XXVIII. Section 243/1998) in conformity with the regulations for animal experimentation in the European Communities Council Directive of 24 November 1986 (86/609/EEC). All experiments were approved by the University of South Florida IACUC and all efforts were made to reduce the number of animals used.

### Synthesis and Formulation of Ketone Precursors

Ketone ester (KE; 1,3-butanediol-acetoacetate diester) was synthesized as previously described (D’Agostino et al., 2013). Ketone salt (KS, which is Na^+^/K ^+^–β-hydroxybutyrate mineral salt) is a novel agent that was mixed into a 50% solution supplying approximately 375 mg/g of pure βHB and 125 mg/g of Na^+^/K^+^ in a 1:1 ratio. Both KE and KS were developed and synthesized in collaboration with Savind Inc. Human food grade medium chain triglyceride (MCT) oil (~60% caprylic triglyceride/40% capric triglyceride) was purchased from Now Foods (Bloomingdale, IL, USA). KS was mixed with MCT in a 1:1 ratio (KSMCT) at the University of South Florida (USF, Tampa, FL, USA).

### Ketone Supplementation

In order to determine the effect of different administration forms, we tested chronic administration, when the ketone supplementation was mixed into the regular rodent chow, which the animals had access to all day for several weeks, and sub-chronic administration when the ketone supplementation was gavaged orally at a single time point daily for only 7 days.

#### Chronic Administration

A total of 48 male SPD rats were fed for 83 days with either standard rodent chow (2018 Teklad Global 18% Protein Rodent Diet (#2018), Harlan) standard diet (SD)/control; *n* = 9) or SD + ketone supplementation. Four treatment animal groups included low-dose KE (~10 g/kg b.w./day, Low-dose ketone ester (LKE); *n* = 10), high-dose KE (~25 g/kg b.w./day, high dose ketone ester (HKE); *n* = 10), KS (~25 g/kg b.w./day, KS; *n* = 9) and KSMCT (~25 g/kg b.w./day, KSMCT; *n* = 10). Higher dose was used for chronic administration, as the rats were consuming food-integrated ketone supplementation throughout the day, not at a single time point.

#### Sub-Chronic Oral Gavage

In order to familiarize the animals to the intragastric gavage method, water was gavaged for 5 days before ketone supplementation. Following the adaptation period to the intragastric gavage method, 39 male SPD rats were fed with standard diet, described in previous studies (Poff et al., 2013) and gavaged daily with 5 g/kg b.w./day water (SD/control; *n* = 11) or ketone supplements KE (*n* = 9), KS (*n* = 9), KSMCT (*n* = 10) sub-chronically for 7 days.

In addition, following the adaptation period to the intragastric gavage method, WAG/Rij male rats (*n* = 32) were fed with SD and gavaged sub-chronically with ~2.5 g/kg b.w./day water (SD/control; *n* = 8), KE (*n* = 8), KS (*n* = 8) or KSMCT (*n* = 8) for 7 days. For the sub-chronic gavage administration the gavage dose was used that induced desired elevation of blood ketone based on our previous studies (Kesl et al., 2016).

### Anxiety Assay

EPM (Coulbourn Instruments) was used to assess anxiety-related behavior of the rats after 83 days of chronic feeding or after 7 days of oral gavage. EPM experiments were carried out under non-stress conditions (in dimly lit and quiet room) between 12.00 h and 14.00 h.

The rats were transferred in their home cage to the experimental room 30 min prior to beginning the experiment. Briefly, rats were placed in the intersection of the four arms of the EPM, facing the open arm opposite to where the experimenter was and their behavior was recorded for 5 min. The amount of time spent and number of entries made on the open arms, closed arms and the center zones were video recorded. Latency to entry into the closed arms and the distance traveled in each zones was also measured in chronically treated SPD rats. Only those behaviors are discussed at each experimental scenario where significant difference was found. At the end of the 5 min test the rats were removed from the maze and placed back into their home cage. The maze was cleaned with 70% alcohol and later with tap water and dried with paper towel between rats. The primary method for data collection was a video-tracking system with computer interface and video camera (SMART V3.0 PLATFORM, Panlab, Harvard Apparatus, Holliston, MA, USA), to automatically collect behavioral data in SPD rats. A blinded observer was present in the testing room separated from the maze by a curtain, and collected EPM data in both SPD and WAG/Rij animals.

### Blood Analyses and Weight Measurement

In the chronic feeding study, blood βHB and glucose levels were measured 24 h before the 1st day of ketone treatments (baseline levels) and at 13th week after the EPM experiment. In the 7 day oral gavage studies, blood βHB and glucose levels were measured 24 h before the 1st day of ketone treatments (baseline levels; SPD and WAG/Rij rats), 24 h after the first gavage and 60 min after gavage on the 7th day (SPD and WAG/Rij rats). Whole blood samples (10 μL) were taken from the saphenous vein for analysis of blood glucose (mg/dl) and βHB (mmol/l) levels with the commercially available glucose and ketone (βHB) monitoring system Precision Xtra^TM^ (Abbott Laboratories, Abbott Park, IL, USA).

The body weight of all animals was recorded before the first ketone treatment (before) and on the last day of the ketone treatment (after).

### Statistics

All data are presented as the mean ± standard error of the mean (SEM). We compared the effects of ketone supplementations on anxiety-related behavior as well as on blood βHB and glucose levels to control or/and baseline levels. Data analysis was performed using GraphPad PRISM version 6.0a. Results were considered significant when *p* < 0.05. Significance was determined by one-way analysis of variance (ANOVA) with Fisher’s LSD test for the behavioral data. Blood ketone, blood glucose and body weight change were compared using a two-way ANOVA with Tukey’s multiple comparisons test.

## Results

### Ketone Supplementation Reduced Anxiety on Elevated Plus Maze

#### More Time Spent in Open Arms with Ketone Supplements

**Figure 1:**
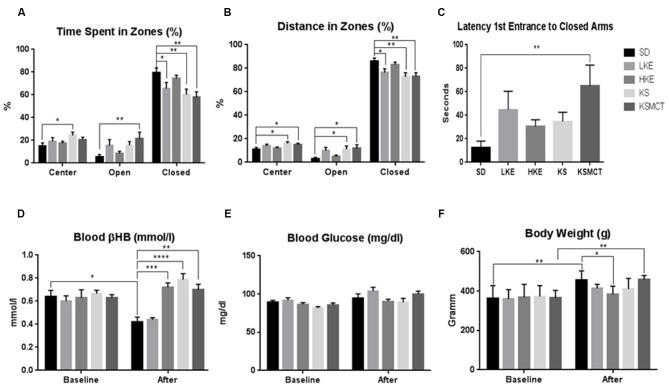
**Response of Sprague-Dawley (SPD) rats to chronic feeding of exogenous ketone supplementation. (A)** Rats consuming KSMCT supplements spent more time in open arms (open), Low-dose ketone ester (LKE), KS and KSMCT groups spent less time in closed arms (closed), showing reduced anxiety compared to control (SD) group; **(B)** Rats consuming ketone supplements traveled more distance in open arms (KS and KSMCT) and less in closed arms (LKE, KS and KSMCT), showing reduced anxiety compared to control group; **(C)** Rats consuming KSMCT entered the closed arms later, showing reduced anxiety compared to control group; **(D)** Rats consuming high dose ketone ester (HKE), KS and KSMCT showed elevated blood ketone levels after 13 weeks (after) compared to control group; **(E)** Blood glucose levels did not change significantly were lower in HKE and KSMCT groups compared to control after 13 weeks; and **(F)** body weight was lower in HKE group after 13 weeks compared to control. Abbreviations: SD, standard rodent chow + water (~25 g/kg b.w. water/day); LKE, SD + LKE (1,3-butanediol-acetoacetate diester, ~10 g/kg b.w./day); HKE, SD + HKE (~25 g/kg b.w./day); KS, SD + beta-hydroxybutyrate-mineral salt (βHB-S; ~25 g/kg b.w./day); KSMCT, SD + βHB-S+medium chain triglyceride (MCT; ~25 g/kg b.w./day); (*p < 0.05; **p < 0.01; ***p < 0.001; ****p < 0.0001).

After chronic feeding of ketone supplementation in SPD rats the time spent in the open arms was significantly more in KSMCT group (*p* = 0.0094), while time spent in the closed arms was significantly less in LKE, KS and KSMCT groups (*p* = 0.0389, 0.0077 and 0.0019, respectively), compared to the control (SD) in SPD rats. Time spent in the center was significantly more in KS group (*p* = 0.0239; Figure 1A).

**Figure 2:**
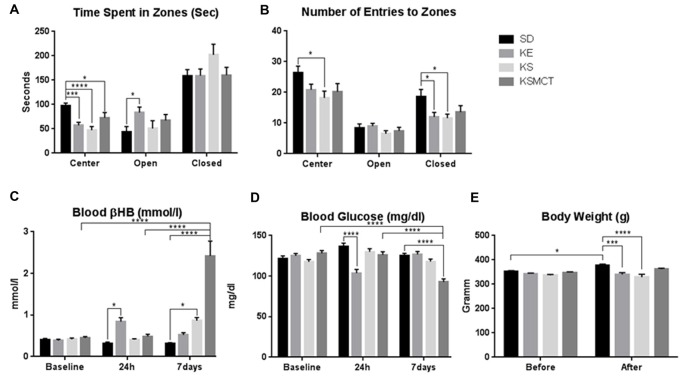
**Response of SPD rats to 7 days oral administration (gavage) of exogenous ketone supplementation. (A)** More time spent in open arms (open) by KE group and less time spent in center by KE, KS and KSMCT groups were detected compared to control; **(B)** Less entries in closed arms (closed) by KE and KS groups; **(C)** Blood βHB levels were higher in KE group after 24 h and in KS and KSMCT groups after 7 days, compared to control; **(D)** Blood glucose levels were lower in KE group after 24 h and in KSMCT group compared to baseline, control and 24 h; **(E)** Body weight was lower in KE and KS groups compared to control after 7 days. Abbreviations: SD, standard rodent chow + water (~5 g/kg b.w. water/day); KE, SD + ketone ester (1,3-butanediol-acetoacetate diester, ~5 g/kg b.w./day); KS, SD + beta-hydroxybutyrate-mineral salt (βHB-S; ~5 g/kg b.w./day); KSMCT, SD + βHB-S + MCT (~5 g/kg b.w./day); (*p < 0.05; ***p < 0.001; ****p < 0.0001).

**Figure 3:**
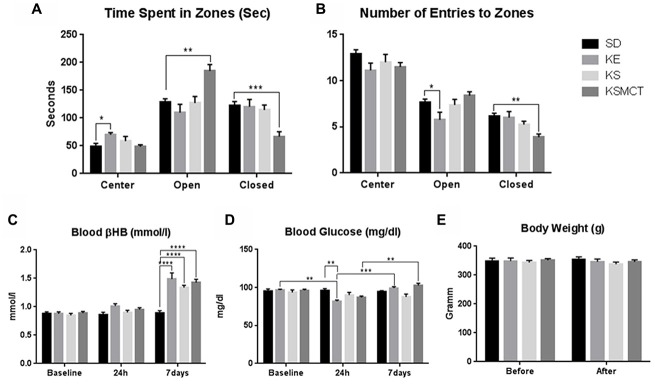
**Response of Wistar Albino Glaxo/Rijswijk (WAG/Rij) rats to 7 days oral administration of exogenous ketone supplementation. (A)** More time spent in open arms (open) and less time spent in closed arms (closed) by KSMCT group were demonstrated compared to control; **(B)** Less entries in closed arms by KSMCT group and in open arms by KE group; **(C)** Blood βHB levels were higher in all treatment groups (KE, KS and KSMCT) after 7 days, compared to baseline, 24h and control. **(D)** Blood glucose levels decreased after 24 h in KE group compared to control and baseline, but increased after 7 days compared to 24 h in KE and KSMCT group; **(E)** Body weight did not change significantly in either groups. Abbreviations: SD, standard rodent chow + water (~2.5 g/kg b.w. water/day); KE, SD + ketone ester (1,3-butanediol-acetoacetate diester, ~2.5 g/kg b.w./day); KS, SD + beta-hydroxybutyrate-mineral salt (βHB-S; ~2.5 g/kg b.w./day); KSMCT, SD + beta-hydroxybutyrate-mineral salt (BHB-S) + MCT (KSMCT; ~2.5 g/kg b.w./day); (*p < 0.05; **p < 0.01; ***p < 0.001; ****p < 0.0001).

After 7 days of gavage administration in SPD rats, the time spent in the open arms increased in the KE group (*p* = 0.0281), whereas time spent in the center decreased in KE, KS and KSMCT groups (*p* = 0.0005, <0.0001 and 0.023, respectively; Figure 2A). In WAG/Rij rats the KSMCT treated rats spent more time in the open arms (*p* = 0.0018) and less time in the closed arms (*p* = 0.0003), whereas KE treated rats spent more time in the center (*p* = 0.0027), compared to the control (SD) group (Figure 3A).

#### Less Entries to Closed Arms with Ketone Supplements

Entries to the closed arms were less frequent with KE and KS treatment (*p* = 0.0436, 0.0234, respectively) in SPD and with KSMCT treatment (*p* = 0.0014) in WAG/Rij rat models, respectively, after 7 days of administration (Figures 2B, 3B). SPD rats also entered fewer times to the center when treated with KS (Figure 2A; *p* = 0.0193), compared to control (SD) animals. Conversely, WAG/Rij rats made less entries to open arms in KE treated group (*p* = 0.0318).

#### More Distance Traveled in Open Arms, Less in Closed Arms and Delayed Latency of Entrance to Closed Arms with Ketone Supplements

After chronic feeding in SPD rats, the distance traveled in the open arms was significantly greater in KS and KSMCT groups (*p* = 0.036 and 0.0165), and distance traveled in the closed arms was significantly less in LKE, KS and KSMCT groups (*p* = 0.0252, 0.00041 and 0.0032), compared to the control (SD). Distance traveled in the center was more in KS and KSMCT groups (*p* = 0.0206 and 0.0482; Figure 1B).

The latency to first entrance of closed arms was significantly greater in KSMCT group after chronic feeding (*p* = 0.0038; Figure 1C).

### Elevation of Blood βHB Levels with Ketone Supplements

After 83 days of chronic feeding in SPD rats, blood βHB levels remained significantly elevated in HKE, KS and KSMCT groups, compared to control (*p* = 0.0004, <0.0001, 0.0014; Figure 1D) while it decreased in SD compared to baseline (*p* = 0.0307).

Blood βHB levels were elevated in SPD rats after 24 h of a single gavage in KE group (Figure 2C; *p* = 0.0325), compared to control.

In SPD rats βHB was elevated in KSMCT groups at 7 days compared to their level at 24 h and baseline (*p* < 0.0001; Figure 2C). Blood βHB was also elevated in KS and KSMCT treatment groups compared to control group (*p* = 0.0194, <0.0001; Figure 2C). After 7 days of gavage, blood βHB was elevated in KE, KS and KSMCT groups in WAG/Rij rats (*p* < 0.0001) compared to baseline, 24 h and control (Figure 3C).

### Ketone Supplementation and Blood Glucose Levels

After 13 weeks of chronic feeding in SPD rats blood glucose did not change significantly in any groups (Figure 1E).

However, in SPD rats, after sub-chronic ketone treatments, blood glucose levels were lower at 24 h in KE group compared to control (*p* < 0.0001; Figure 2D). After 7 days of oral gavage blood glucose was lower in KSMCT compared to control, to baseline and to the level at 24 h in SPD rats (*p* < 0.0001; Figure 2D).

In WAG/Rij rats the KE group had lower glucose levels after 24 h, compared to baseline levels (*p* = 0.0064), however after 7 days their level were elevated again, compared to the level at 24 h (*p* = 0.0006; Figure 3D). Moreover, glucose levels were also elevated after 7 days compared to 24 h in KSMCT group (Figure 3D).

### Differences in Changes of Blood Ketone and Glucose Levels Between the Two Animal Models

**Figure 4:**
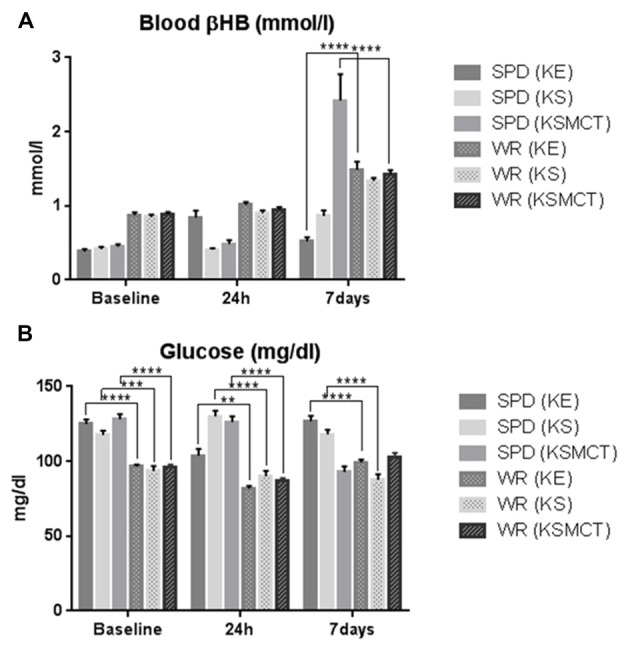
**Blood βHB and glucose levels compared between the two animal models. (A)** Blood βHB levels were higher at 7 days in WAG/Rij rats after KE and KSMCT treatment. **(B)** Blood glucose levels were significantly lower in WAG/Rij rats, except in KSMCT group at 7 days. (**p < 0.01; ***p < 0.001; ****p < 0.0001).

There was significant difference in βHB levels between the two animal models in KE and KSMCT groups at 7 days only (Figure 4A). The glucose levels were different between the two animal models in each treatment groups at each time points, except in KSMCT group at 7 days (Figure 4B).

### Body Weight Changes during Ketone Supplementation

After chronic feeding, the body weight of SPD rats was lower in HKE group compared to the control (*p* = 0.0366). The body weight increased in SD and KSMCT groups, compared to their baseline (*p* = 0.0015, 0.0012; Figure 1F).

After 7 days of treatment the body weight of SPD rats increased in SD group, compared to its baseline (*p* = 0.0297). The body weight was lower in KE and KS treatment groups after 7 days, compared to control (*p* = 0.0005, <0.0001; Figure 2E). In WAG/Rij rats the body weight did not change significantly in either group during the treatment period (Figure 3E).

## Discussion

The current study demonstrated the anxiolytic effect of chronic (13 weeks) and sub-chronic (7 days) administration of several forms of ketone supplementation in both SPD and WAG/Rij rats. Anxiolytic effect was assessed by means of EPM and measured by less entries and time spent in closed arms, more entries and time spent in open arms, more distance traveled in open arms, and delayed latency to entrance into closed arms.

Ketones are produced naturally in the liver only under certain physiological conditions associated with the suppression of the hormone insulin: starvation, fasting, calorie restriction, prolonged exercise, or during the consumption of high fat, low carbohydrate KD. The restrictive nature of these states has limited the clinical applicability of therapeutic ketosis due to practical considerations. In an effort to circumvent this dilemma, researchers have recently developed a number of exogenous ketogenic supplements, ketogenic precursors that are metabolized to produce a dose-dependent elevation of βHB and AcAc in the blood (Veech, 2004; Clarke et al., 2012; Kesl et al., 2016). Induction of hyperketonemia produces acute and chronic changes in metabolic physiology and neuropharmacological pathways that provide therapeutic effects in varied disease states. The ketone supplements tested in this study allowed for a rapid and controlled induction of physiologic ketosis without the need for fasting or severe dietary restrictions. Previous studies have demonstrated the use of exogenous ketones as a means to induce a dose-dependent hyperketonemia (1–7 mM) in rats, mice, dogs, pigs and humans (Desrochers et al., 1992, 1995; Ciraolo et al., 1995; Brunengraber, 1997; Puchowicz et al., 2000; Clarke et al., 2012; Srivastava et al., 2012). Exogenous ketogenic supplementation mimics the metabolic and physiologic effects of the KD, including enhancing mitochondrial biogenesis, anaplerosis, suppression of glycolysis and increasing ATP and adenosine production, all thought to mediate the therapeutic effects of KD in epilepsy (Veech, 2004; Srivastava et al., 2012; Kesl et al., 2014; Poff et al., 2015). Since many of the benefits of ketosis are mechanistically attributable to the ketone bodies, it is possible that exogenous ketone supplementation could mimic the therapeutic efficacy of the KD for certain disorders, including anxiety disorders, or other disorders, such as seizure disorders and AD that have a comorbidity of anxiety.

The differences in blood βHB and glucose levels between the two animal models both before and after the treatment highlight the need to examine the response to ketone supplementation in more rodent models with different pathologies. Both in chronic and sub-chronic KE treatment resulted in lower body weight in SPD animals, however, the body weight did not change significantly during the treatment period in WAG/Rij rats.

Previous studies have shown a clear anxiolytic effect in the EPM when the antidepressant/antipanic drug phenelzine, agonists and/or antagonists of different neurotransmitter systems (e.g., GABAergic and glutamatergic system) were given acutely to rats (Paslawski et al., 1996; Engin and Treit, 2008). Although, there is no compelling evidence that the KD or elevated blood ketone levels would induce global changes in GABA levels (Hartman et al., 2007), the exogenous ketone supplementation tested in the present study similarly increased the open arms exploration in the EPM and increased the latency to enter the closed arms, showing anxiolytic effect.

In summary, LKE decreased time spent in closed arms and reduced distance traveled in closed arms after chronic treatment. Moreover, KE increased time spent in open arms, decreased time spent in center and decreased number of entries in closed arms after sub-chronic treatment in SPD rats. KS was proven to be effective in reducing time spent and distance traveled in closed arms and increase distance traveled in open arms after chronic treatment. It also reduced time spent and number of entries to center, while decreasing number of entries in closed arms after sub-chronic treatment in SPD rats. KSMCT effectively increased time spent and distance traveled in open arms and decreased time spent and distance traveled in closed arms, as well as delayed latency to first entrance to closed arms after chronic treatment in SPD rats. In WAG/Rij rats KSMCT successfully increased time spent in open arms and decreased time spent and number of entries in closed arms after sub-chronic treatment. Differences could be observed between the effects of different ketone supplements on anxiety. These results indicate that KS and KSMCT are the most effective after chronic treatment, while KE and KS seem to be the most effective after sub-chronic treatment in rats without pathology (SPD). In rats with pathology (WAG/Rij) KSMCT was the most effective treatment after sub-chronic administration.

Previous studies showed that the percentage of open arm entries linearly increase with age (Lynn and Brown, 2010) and the aging-related changes in EPM behavior are strain-specific (Ferguson and Gray, 2005), therefore the rats used in the present study involved two strains and animals of different age. In other studies, those rats that were pre-treated with amphetamine exhibited increased anxiety-like behavior on the EPM, which was successfully reversed by paroxetine, a selective serotonin (5-HT) reuptake inhibitor (Tu et al., 2014). Those results suggested that 5-HT levels in the ventral hippocampus are critical for regulating anxiety behavior and that increasing 5-HT levels may be an effective strategy for reducing anxiety (Tu et al., 2014). Therefore, we speculate that the ketone supplements reduced anxiety-related behavior not solely by elevating blood ketone levels, but may also effect the regulation of 5-HT levels. In spite of that WAG/Rij rats show different anxiety behavior compared to SPD rats (e.g., WAG/Rij rats spent approximately equal times in the closed and open arms without ketone supplementation: Figures 2A, 3A), sub-chronic ketone supplementation was effective not only in SPD rats but also in WAG/Rij rats (Figures 2A,B, 3A,B). Thus, our results on WAG/Rij rats strengthened the hypothesis on anxiolytic effect of exogenous ketone supplementation found in SPD rats.

Previous studies demonstrated that 18% of Americans and 14% of Europeans may be affected by one or more anxiety disorders (Kessler et al., 2005) that are associated with high financial costs, increased risk of mortality and morbidity as well as impaired workplace performance (Greenberg et al., 1999; Albert et al., 2005). Ketone supplementation may be a potential therapeutic intervention in treatment of anxiety disorders (Yudkoff et al., 2007; Engin and Treit, 2008; Masino et al., 2012; Lutas and Yellen, 2013), while very little is known about the link between ketone application-evoked changes in CNS and anxiety disorders. However, it has been demonstrated that KD may: (i) decrease extracellular glutamate release/level by means of inhibition of vesicular glutamate transporter; (ii) increase adenosine level; and (iii) augment the GABAergic effects by GABA_A_ receptors (Yudkoff et al., 2007; Engin and Treit, 2008; Masino et al., 2012; Lutas and Yellen, 2013). It has been demonstrated that serotonergic, glutamatergic and GABAergic system of different brain areas such as hippocampus and/or amygdala have a role in the regulation of anxiety: 5-HT transporters, 5-HT receptors (e.g., 5-HT1A), N-methyl-D-aspartate (NMDA) receptors and GABA receptors (e.g., GABA_A_ receptors) are potential targets in the treatment of anxiety disorders (Nagy et al., 1979; Kakui et al., 2009; Li, 2012; Sankar, 2012; Dias et al., 2013). It was concluded that GABAergic system may have critical role in the modulation of the level of anxiety: increased GABAergic transmission may evoke anxiolytic effect (Engin and Treit, 2008; Li, 2012). Thus, augmentation of the GABAergic effects by means of KD *via* GABA_A_ receptors may evoke a decrease in anxiety level. It has also been demonstrated that: (i) KD may increase extracellular adenosine level (Masino et al., 2012; Lutas and Yellen, 2013); (ii) inhibition of adenosine receptors (A_1_R and A_2A_R) by means of caffeine promotes anxious behavior (Klein et al., 1991); (iii) A_1_R- or A_2A_R-knockout mice showed anxiogenic-like behaviors (Ledent et al., 1997; Johansson et al., 2001); and (iv) modulation of adenosine receptor activity might be an effective treatment strategy for patients with anxiety disorders (Kovács and Dobolyi, 2013). In addition, as KD may evoke decreased extracellular glutamate level (Lutas and Yellen, 2013) and NMDA receptor antagonists may have anxiolytic effects (Guimarães et al., 1991; Engin and Treit, 2008) KD may exert its alleviating effect on anxiety level *via* glutamatergic system. Thus, theoretically, as ketone supplementation may generate similar changes in brain neurotransmitter systems as KD by means of ketosis (Figures 1–3), chronic and sub-chronic ketone supplementation-provoked anxiolytic effects may be evoked by means of glutamatergic and/or GABAergic as well as adenosinergic system in SPD and WAG/Rij rats. Indeed, a recent study supports the effect of ketone esters increasing the brain GABA/Glutamate ratio in an animal model of Angelman’s syndrome (Ciarlone et al., 2016). However, our knowledge is not sufficient at present to explain the mechanism(s), by which ketone supplementation exerts its anti-anxiety effects.

We measured higher βHB levels after sub-chronic ketone supplementation in WAG/Rij rats (KE and KSMCT) compared to SPD rats (Figure 4A). This result and the reduced activity of GABAergic system in WAG/Rij rat brain (Luhmann et al., 1995) may explain that half doses of KE and KSMCT (~2.5 g/kg b.w./day) than applied in SPD rats (~5 g/kg b.w./day) effectively decreased the anxiety level in WAG/Rij rats. Higher basal ketone levels (and its putative anti-anxiety effects) in WAG/Rij rats may also cause lower basal anxiety levels compared to SPD rats (e.g., WAG/Rij rats spent more time in the open arms compared to SPD rats before ketone supplements; Figures 2A, 3A).

*In conclusion*, based on the present study, we can conclude that chronic and sub-chronic administration of exogenous ketone supplementation may be an effective way to reduce anxiety. Achieving nutritional ketosis with exogenous ketone supplementation while maintaining a normal diet might be an alternative to the KD, or may further augment the therapeutic efficacy of the KD. Therefore, it is important to understand the long-term effects of these supplements fed chronically. These preliminary data show that chronic and sub-chronic feeding of ketone supplements not only elevated blood ketone levels, but also reduced anxiety-related behavior, which can be highly beneficial for patients managing diseases like epilepsy and AD with nutritional ketosis. Since achieving nutritional ketosis requires strict dietary restrictions, compliance is a major difficulty in this treatment. The administration of exogenous ketone supplements that increase ketone levels in the blood without dietary restrictions may be an effective option to improve compliance. We propose that exogenous ketone supplementation could provide an alternative method to reduce anxiety for healthy individuals, as well as those with disorders that are metabolically managed with the KD. Therefore, further studies are needed to determine the molecular basis of ketone supplementation-induced anxiolytic changes, and how this strategy can be implemented in human clinical trials with patients suffering anxiety disorders.

## Author Contributions

CA: conception and design of experiments, data collection, analysis and interpretation of data, writing manuscript; ZK: design of experiments, data collection, analysis and interpretation of data, writing manuscript; APK, AMP and SLK: data collection, revising manuscript; CM and CRG: data collection; GJ and DPD: design of experiments, revising manuscript.

## Conflict of Interest Statement

International Patent # PCT/US2014/031237, University of South Florida, D.P. D’Agostino, S. Kesl, P. Arnold, “*Compositions and Methods for Producing Elevated and Sustained Ketosis*”. P. Arnold (Savind) has received financial support (ONR N000140610105 and N000140910244) from D.P. D’Agostino (USF) to synthesize ketone esters. Provisional patent # 16A007, University of South Florida, C. Ari, P. Arnold, D.P. D’Agostino “*Exogenous Ketone Supplements for Reducing Anxiety-Related Behavior*”. The other authors declare that the research was conducted in the absence of any commercial or financial relationships that could be construed as a potential conflict of interest. The reviewer DR and handling Editor declared their shared affiliation, and the handling Editor states that the process nevertheless met the standards of a fair and objective review.

## Abbreviations

5-HT: Serotonin
AD: Alzheimer’s disease
βHB: beta-hydroxybutyrate
CNS: central nervous system
EPM: elevated plus maze
GLUT1 DS: Glucose transporter type-1 deficiency syndrome
HKE: high-dose ketone ester
KD: ketogenic diet
LKE: low-dose ketone ester
MCT: medium chain triglyceride
SD: standard diet
SPD rats: Sprague-Dawley rats
WAG/Rij rats: Wistar Albino Glaxo/Rijswijk rats.
