# Corrigendum: Exogenous Ketone Supplements Reduce Anxiety-Related Behavior in Sprague-Dawley and Wistar Albino Glaxo/Rijswijk Rats

**DOI:** 10.3389/fnmol.2017.00036

**Published:** 2017-02-13

**Authors:** Csilla Ari, Zsolt Kovács, Gabor Juhasz, Cem Murdun, Craig R. Goldhagen, Andrew P. Koutnik, Angela M. Poff, Shannon L. Kesl, Dominic P. D'Agostino

**Affiliations:** ^1^Department of Molecular Pharmacology and Physiology, Hyperbaric Biomedical Research Laboratory, Morsani College of Medicine, University of South FloridaTampa, FL, USA; ^2^Department of Zoology, University of West HungarySzombathely, Hungary; ^3^Proteomics Laboratory, Eotvos Lorand UniversityBudapest, Hungary

**Keywords:** anxiety, exogenous ketone supplements, ketones, elevated plus maze, animal models

An author name was incorrectly spelled as Andrew M. Koutnik. The correct spelling is Andrew P. Koutnik. The authors apologize for this error and state that this does not change the scientific conclusions of the article in any way. The original article has been updated.

## Conflict of interest statement

International Patent # PCT/US2014/031237, University of South Florida, DD, SK, P. Arnold, “Compositions and Methods for Producing Elevated and Sustained Ketosis.” P. Arnold (Savind) has received financial support (ONR N000140610105 and N000140910244) from DD (USF) to synthesize ketone esters. Provisional patent # 16A007, University of South Florida, CA, P. Arnold, DD “Exogenous Ketone Supplements for Reducing Anxiety-Related Behavior.” The other authors declare that the research was conducted in the absence of any commercial or financial relationships that could be construed as a potential conflict of interest.

